# Valacyclovir and Acyclovir Are Substrates of the Guanine Deaminase Cytosolic PSD‐95 Interactor (Cypin)

**DOI:** 10.1002/prot.26740

**Published:** 2024-08-29

**Authors:** Keith R. Lange, Noor Rasheed, Xiaoyang Su, M. Elena Diaz‐Rubio, Bonnie L. Firestein

**Affiliations:** ^1^ Department of Cell Biology and Neuroscience Rutgers, The State University of New Jersey Piscataway New Jersey USA; ^2^ Graduate Program in Biochemistry Rutgers, The State University of New Jersey Piscataway New Jersey USA; ^3^ Department of Medicine Robert Wood Johnson Medical School, Rutgers, The State University of New Jersey New Brunswick New Jersey USA; ^4^ Rutgers Metabolomics Shared Resource, Cancer Institute of New Jersey New Brunswick New Jersey USA

**Keywords:** Amplex Red assay, guanine deaminase, Michaelis–Menten kinetics, molecular dynamics, NADH‐coupled assay, tryptophan fluorescence

## Abstract

Valacyclovir, enzymatically hydrolyzed in the body to acyclovir, is a guanine‐based nucleoside analog commonly prescribed as an antiviral therapy. Previous reports suggest that guanosine analogs bind to guanine deaminase; however, it is unclear whether they act as inhibitors or substrates. Data from our laboratory suggest that inhibition of guanine deaminase by small molecules attenuates spinal cord injury‐induced neuropathic pain. Here, we examine whether the guanosine analogs valacyclovir and acyclovir are deaminated by cypin (**cy**tosolic **P**SD‐95 **in**teractor), the major guanine deaminase in the body, or if they act as cypin inhibitors. Using purified *Rattus norvegicus* cypin, we use NADH‐coupled assay to confirm deamination of valacyclovir and determined Michaelis–Menten constants. Subsequently, we use tryptophan fluorescence quenching assay to calculate dissociation constants for valacyclovir and acyclovir and find that inclusion of the valine motif in valacyclovir increases affinity for cypin compared to acyclovir. To our knowledge, neither *K*_m_ nor *K*_D_ values for cypin has been previously reported for either compound. We use Amplex Red assay and demonstrate that both valacyclovir and acyclovir are cypin substrates and that their metabolites are further processed by xanthine oxidase and uricase. Using molecular dynamics simulations, we demonstrate that an alpha helix near the active site is displaced when valacyclovir binds to cypin. Furthermore, we used LC–MS‐based assay to directly confirm deamination of valacyclovir by cypin. Taken together, our results demonstrate a novel role for cypin in deamination of valacyclovir and acyclovir and suggest that therapeutics based on purine structures may be inactivated by cypin, decreasing inhibitory efficacy.

## 1 Introduction

Cypin, **cy**tosolic **P**SD‐95 **in**teractor, is a 50 kDa enzyme that promotes dendritic arborization in hippocampal neurons [1, 2, 3, 4, 5] and is the primary guanine deaminase in the body [6, 7, 8]. Cypin catalyzes the deamination of guanine to xanthine using coordinated Zn^2+^ in its active site [3], and xanthine is further converted to uric acid, the end product of purine metabolism in humans. Given that approximately 80% of xanthine produced is derived from guanine metabolism [9], it is expected that inhibition of cypin would decrease the levels of uric acid, which may serve as a potential therapeutic for hyperuricemia or gout, where serum levels of uric acid are >7.0 mg/dL in adults [10].

Valacyclovir is a guanine‐based nucleoside analog that has been prescribed as an antiviral therapy for nearly three decades [11, 12]. Valacyclovir, usually administered orally, is enzymatically hydrolyzed to the pharmacologically active drug acyclovir [11]. Once transported across the membrane, acyclovir is targeted by viral thymidylate kinases, which ultimately attenuates the rate of viral replication through DNA chain termination. Acyclovir is eliminated from the body mostly unchanged; however, there are two known metabolites, 8‐hydroxy‐acyclovir (1% conversion) and [(carboxymethoxy)methyl]guanine (<15% conversion) [13].

It was recently reported that guanosine analogs, such as valacyclovir and acyclovir, have detectable affinity for cypin as indicated by protein thermal shift assays and a high‐resolution crystal structure (1.99 Å) of the cypin‐valacyclovir complex [14]. However, it is unknown whether valacyclovir acts as an inhibitor or a non‐endogenous substrate of cypin. In addition, an assessment of the crystallization data demonstrates that there is lower electron density for both the modeled valacyclovir molecule and the cypin residues that make contact with valacyclovir, suggesting partial dissociation of valacyclovir from cypin [14]. Furthermore, there are several amino acids within a hydrophobic pocket of cypin adjacent to the active site [14]. Thus, we used biochemical and computational assays to determine whether valacyclovir and acyclovir can be metabolized by the purine metabolism pathway and to detect conformational differences between valacyclovir‐bound and apo structure models of cypin. Our results suggest that valacyclovir and acyclovir are substrates of cypin, and their metabolites can be further processed by xanthine oxidase and uricase.

## 2 Materials and Methods

### 2.1 Expression and Purification of Cypin, the Major Guanine Deaminase

pET15B vector containing cDNA encoding cypin from *Rattus norvegicus* was transformed into BL21 *Escherichia coli*, and resulting colonies were stored as glycerol stocks. Starter cultures were grown in Terrific Broth (TB) + 1% v/v glycerol and 50 μg/mL kanamycin for 16 h at 37°C with shaking at 200 rpm. The following day, starter cultures were diluted 50‐fold in 1 L TB + 1% v/v glycerol and 50 μg/mL kanamycin and incubated at 37°C with shaking at 200 rpm until the OD_600_ was ≥1.0. Isopropyl β‐D‐1‐thiogalactopyranoside (IPTG) was added to a final concentration of 1 mM IPTG, and cultures were incubated at 37°C with shaking at 200 rpm for 5 h. Bacteria were pelleted by centrifugation at 4600 × *g* for 30 min at 4°C. Bacterial pellets were then lysed in 6 mL lysis buffer (100 mM Tris pH 7.4, 400 mM NaCl, 10% v/v glycerol, 1 mM DTT, 1% v/v Triton X‐100, 1 mM PMSF, 20 μg/mL DNAse 1, and 1× complete Mini EDTA‐free protease; Sigma cat#: 11836170001) per 1 g bacterial pellet. The bacterial extract was sonicated on ice at 20% amplitude for 5 min with 1 s on/off pulses. Afterwards, the lysate was rocked at 4°C for 25 min and centrifuged at 20 000 × *g* at 4°C. The supernatant containing Cypin‐6xHis was poured over a gravity column containing 1 mL Ni‐NTA resin pre‐equilibrated with EQ buffer (100 mM Tris pH 7.4, 400 mM NaCl, 10 mM imidazole, and 10% v/v glycerol). The Ni‐NTA resin was washed with at least 2–3 column volumes of washing buffer (100 mM Tris pH 7.4, 400 mM NaCl, 35 mM imidazole, and 10% v/v glycerol) until no protein eluted from the as column indicated by a Bradford test (80 μL Bradford Reagent +20 μL eluate). Bound protein was eluted with 15 mL elution buffer (100 mM Tris pH 7.4, 400 mM NaCl, 400 mM imidazole, and 10% v/v glycerol), and eluate was concentrated with buffer exchange into storage buffer (100 mM Tris pH 7.4, 400 mM NaCl, and 10% v/v glycerol) using a PD10 desalting column. Purified fractions were concentrated to ~3.0 mg/mL and stored at −80°C for future use in experiments.

### 2.2 Determination of *K*_m_

Determination of Michaelis–Menten constants (*K*_m_) was achieved using the NADH‐coupled assay, summarized here. Briefly, recombinant cypin was diluted to 400 nM in 100 mM sodium phosphate, 25 mM NaCl, 200 μM reduced nicotinamide adenine dinucleotide (NADH), 1 mM α‐ketoglutarate, and 4 U/well L‐glutamic dehydrogenase (Sigma cat#: G2626). Reactions in the presence of guanine were initiated by combining 10 μL guanine at the noted concentration dissolved in 0.1 M NaOH and 190 μL reaction mix. For IC_50_ experiments, valacyclovir and acyclovir were diluted in reaction mix and guanine concentration was maintained at 50 μM. Valacyclovir reactions were initiated by combining 100 μL valacyclovir at the noted concentration dissolved in water and 100 μL reaction mix. Reactions were measured over time using a Varioskan LUX multimode microplate reader. The initial rate of deamination was taken within the first 20 s of adding guanine or valacyclovir and computed by measuring the decrease in absorbance at 340 nm over time as NADH is consumed. Resulting velocities of the deamination of guanine and valacyclovir reactions were plotted in GraphPad Prism. Guanine deamination velocities were fitted with nonlinear regression using the Michaelis–Menten model; however, valacyclovir deamination velocities were fitted with nonlinear regression using an allosteric sigmoidal model (*h* = 1.2) due to apparent positive cooperativity. Average *K*_m_ values were taken from three independent trials.

### 2.3 Determination of *K*_D_

The dissociation constants (*K*_D_) for guanine, valacyclovir, and acyclovir were determined using a tryptophan fluorescence quenching assay. Ligands were titrated into 1300 μL buffer (20 mM HEPES pH 7.0, 100 mM NaCl, and 0.5 mM EDTA) containing 4 μM purified cypin at room temperature. Fluorescence values were recorded using the Varioskan LUX multimode microplate reader with the cuvette‐cradling μDrop adapter (ex: 290 nm, em: 345–350 nm, 10 mm pathlength). Dilutions were performed by adding 1 μL increments of ligand to a quartz cuvette containing cypin in buffer and were allowed to equilibrate for 15 s before taking measurements. In total, 30 ligand concentrations were titrated and analyzed. Specific changes in fluorescence were determined by correction for the inner filter effect using 20 μM N‐acetyl‐L‐tryptophanamide (NATA) and plotted against ligand concentrations in GraphPad Prism using the one site‐specific binding method of nonlinear regression.

### 2.4 Xanthine Oxidase and Uric Acid Activity of Deaminated Valacyclovir

To determine whether cypin‐promoted deamination products of guanosine analogs are further substrates of purine metabolism, we used an Amplex Red assay to measure xanthine oxidase activity (Thermofisher cat#: A22182) and uricase activity (Thermofisher cat#: A22181). Prior to Amplex Red assay, dilutions of valacyclovir and acyclovir were incubated under conditions containing either no enzyme, cypin only (500 nM), or cypin and xanthine oxidase (500 nM and 0.04 U/mL, respectively) in 100 mM Tris–HCl pH 7.5, 25 mM NaCl buffer. Reactions were incubated at 37°C for 4–6 h. For assessment of xanthine oxidase and uricase activity, the previous reaction mix was mixed 1:1 with buffer containing 100 mM Tris pH 7.5, 10 μM Amplex Red reagent, and either 0.04 U/mL xanthine oxidase or 0.4 U/mL uricase. Reactions were incubated at 37°C for 30 min, and absorbance at 560 nm for all conditions was recorded using the Varioskan LUX multimode microplate reader.

### 2.5 LC–MS Analysis of Valacyclovir Deamination

To directly measure the deamination and subsequent processing of valacyclovir by the cypin and xanthine oxidase, an LC–MS‐based method was used. Cypin (1 μM) was dissolved in 20 mM HEPES pH 7.0, 25 mM NaCl containing 0.04 U/mL xanthine oxidase. Control conditions were carried out in the absence of cypin and xanthine oxidase. Reactions were initiated by combining 10 μL valacyclovir (2.5 mM final concentration) with 190 μL buffer containing cypin and xanthine oxidase. Reactions were stopped after 2 h incubation at 37°C with 23 μL 10% v/v formic acid.

The LC–MS analysis was performed on a Q Exactive PLUS hybrid quadrupole‐orbitrap mass spectrometer coupled to a Vanquish Horizon UHPLC system (Thermo Fisher Scientific, Waltham, MA) with an XBridge BEH Amide column (150 mm × 2.1 mm, 2.5 μm particle size, Waters, Milford, MA). The HILIC separation used a gradient of solvent A (95%:5% H_2_O:acetonitrile with 20 mM acetic acid, 40 mM ammonium hydroxide, pH 9.4) and solvent B (20%:80% H_2_O:acetonitrile with 20 mM acetic acid, 40 mM ammonium hydroxide, pH 9.4). The gradient was 0 min, 100% B; 3 min, 100% B; 3.2 min, 90% B; 6.2 min, 90% B; 6.5 min, 80% B; 10.5 min, 80% B; 10.7 min, 70% B; 13.5 min, 70% B; 13.7 min, 45% B; 16 min, 45% B; 16.5 min, 100% B; and 22 min, 100% B [15]. The flow rate was 300 μL/min. The column temperature was set to 25°C. The autosampler temperature was set to 4°C, and the injection volume was 5 μL. MS scans were obtained in negative ionization mode with a resolution of 70 000 at *m*/*z* 200, in addition to an automatic gain control target of 3 × 10^6^ and *m*/*z* scan range of 72–1000. The RAW MS data were processed using QualBrowser (Thermo Fisher Scientific) to generate the extracted ion chromatograms and the MS spectra.

### 2.6 Molecular Dynamics Simulations

To explore the dynamics of the apo structure and of different conformational states of cypin bound to valacyclovir, we performed molecular dynamics simulations to predict how each cypin residue moves over time [16]. These simulations were performed on a valacyclovir‐bound model (PDB: 4AQL) and an apo structure model (PDB: 2UZ9) using Gromacs version 2021.2. Given that no crystal structures are available for cypin, we excluded the xanthine ligand in the original modeled structure to produce the apo structure. All water and ligand molecules were removed except valacyclovir. Topology files for the valacyclovir ligand were prepared using the official server for CHARMM general forcefield, CGenFF. The topology of the protein constituents of the simulation were generated with the pdb2gmx command of the CHARMM36 forcefield. The models were centered in a dodecahedron box with a 1 nm boundary from the edge of the box. The box was solvated with the SPC/E water model, and 16 Cl atoms were added to the box to neutralize the charge of the system. The systems of both complexes were equilibrated at 300 K for 100 ps as the NVT ensemble. Similarly, both systems were equilibrated at 1 atm pressure for 100 ps. The molecular dynamics simulation was carried out for 10 ns, and resulting trajectories were visualized in PyMOL.

## 3 Results

### 3.1 Determination of *K*_m_ Values for Guanine and Valacyclovir Using NADH‐Coupled Assay

**FIGURE 1:**
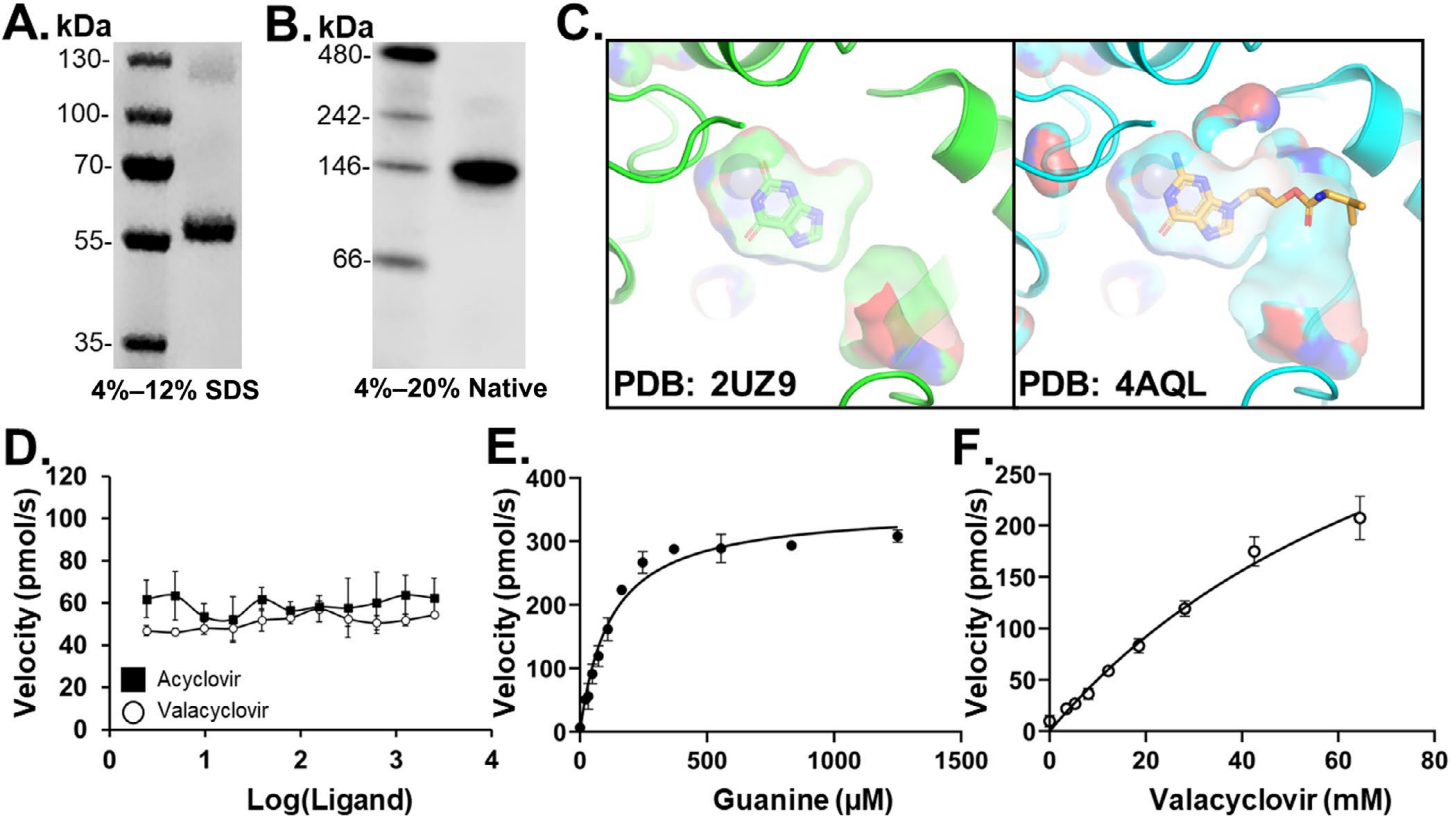
The guanosine analog valacyclovir is a substrate of recombinant guanine deaminase from *Rattus norvegicus*. (A) Recombinant cypin with an N‐terminal 6x‐His tag was purified using a single step purification protocol using Ni‐NTA resin. Cypin is detected at the correct size, ~51 kDa + His tag, when resolved on a 4%–12% Bis‐Tris SDS‐polyacrylamide gel. (B) Recombinant cypin is a homodimer. Inclusion of a thrombin cleavage site and 6xHis‐tag causes the 110 kDa homodimer to run at a higher molecular weight on a 4%–20% native polyacrylamide gel. (C) Difference in cavity space in xanthine‐bound (green) and valacyclovir‐bound (cyan) human cypin models after alignment of both models in PyMOL. (D) Failed IC_50_ determination using the NADH‐coupled assay with 50 μM guanine and 0–2.5 mM valacyclovir or acyclovir. (E) Determination of *K*_m_ for deamination of guanine by cypin using the NADH‐coupled assay. The reaction contains 400 nM recombinant cypin, 4 units glutamate dehydrogenase, 200 μM NADH, 1 mM α‐ketoglutarate, 100 mM phosphate pH 7.4, 100 mM NaCl, pH 7. (F) Determination of *K*_m_ for deamination of valacyclovir by cypin using the NADH‐coupled assay. Error bars represent mean ± SD.

Previous work suggests that nucleoside analogs, namely valacyclovir, acyclovir, penciclovir, and ganciclovir, bind to cypin in a manner similar to that of the endogenous substrate guanine. To determine whether cypin can deaminate valacyclovir, we used an NADH‐coupled assay, where ammonia produced by the cypin‐dependent deamination reaction drives the oxidation of NADH by glutamate dehydrogenase [17]. Purified cypin enzyme, with a typical yield of 5–8 mg per liter of culture, was used for the NADH‐coupled assay. We validated that our recombinant cypin is pure and adopts the expected dimer structure as observed on native polyacrylamide gels (Figure 1A,B).

Comparison of the hydrophobic cavities in and around the active site between xanthine‐bound and valacyclovir‐bound cypin models suggests a significant conformational change in human cypin when valacyclovir is bound (Figure 1C). To assess if valacyclovir or acyclovir inhibits the guanine deaminase activity of cypin, we conducted IC_50_ experiments and observed that neither compound slowed the rate of deamination (Figure 1D). Using the NADH‐coupled assay, we determined *K*_m_ values for guanine and valacyclovir. Although we attempted to determine *K*_m_ and *V*_max_ values for acyclovir, we were unable to accurately do so due to solubility issues. We determined a *K*_m_ value for guanine to be 0.120 ± 0.023 mM and for valacyclovir to be 54.6 ± 13.0 mM, respectively (Figure 1E,F and Table 1). Using calculated *V*_max_ values, we determined the *k*_cat_ values for guanine as 4.43 ± 0.27 s^−1^ and the *k*_cat_ value for valacyclovir as 4.83 ± 0.87 s^−1^. Furthermore, we calculated specificity constants (*k*_cat_/*K*_m_) for guanine and valacyclovir as 36 844 ± 2266 and 88.4 ± 5.9 s^−1^ M^−1^, respectively. The fold change between the specificity constants of guanine and valacyclovir was calculated to be 422 ± 56. These results are consistent with reported structures of the valacyclovir‐bound (PDB: 4AQL) and xanthine‐bound (PDB: 2UZ9) crystal structures of cypin, revealing that binding of valacyclovir significantly changes the substrate binding cavity of cypin to include solvent‐exposed residues.

**TABLE 1:** *K*_m_ and *V*_max_ values of guanine and valacyclovir as determined by the NADH‐coupled guanine deaminase assay.

	Trial 1		Trial 2		Trial 3		Average (*x̄*)		Standard deviation (*σ*_*x*_)	
	*K*_m_ (mM)	*V*_max_ (pmol/s)	*K*_m_ (mM)	*V*_max_ (pmol/s)	*K*_m_ (mM)	*V*_max_ (pmol/s)	*K*_m_ (mM)	*V*_max_ (pmol/s)	*K*_m_ (mM)	*V*_max_ (pmol/s)
Guanine	0.0936	328.6	0.126	365.3	0.139	367.2	0.120	353.7	0.023	21.74
Valacyclovir	41.5	306.1	67.4	427.7	54.8	425.1	54.6	386.3	13.0	69.44

### 3.2 Determination of *K*_D_ Values of Guanine, Acyclovir, and Valacyclovir Using Tryptophan Fluorescence Assay

**FIGURE 2:**
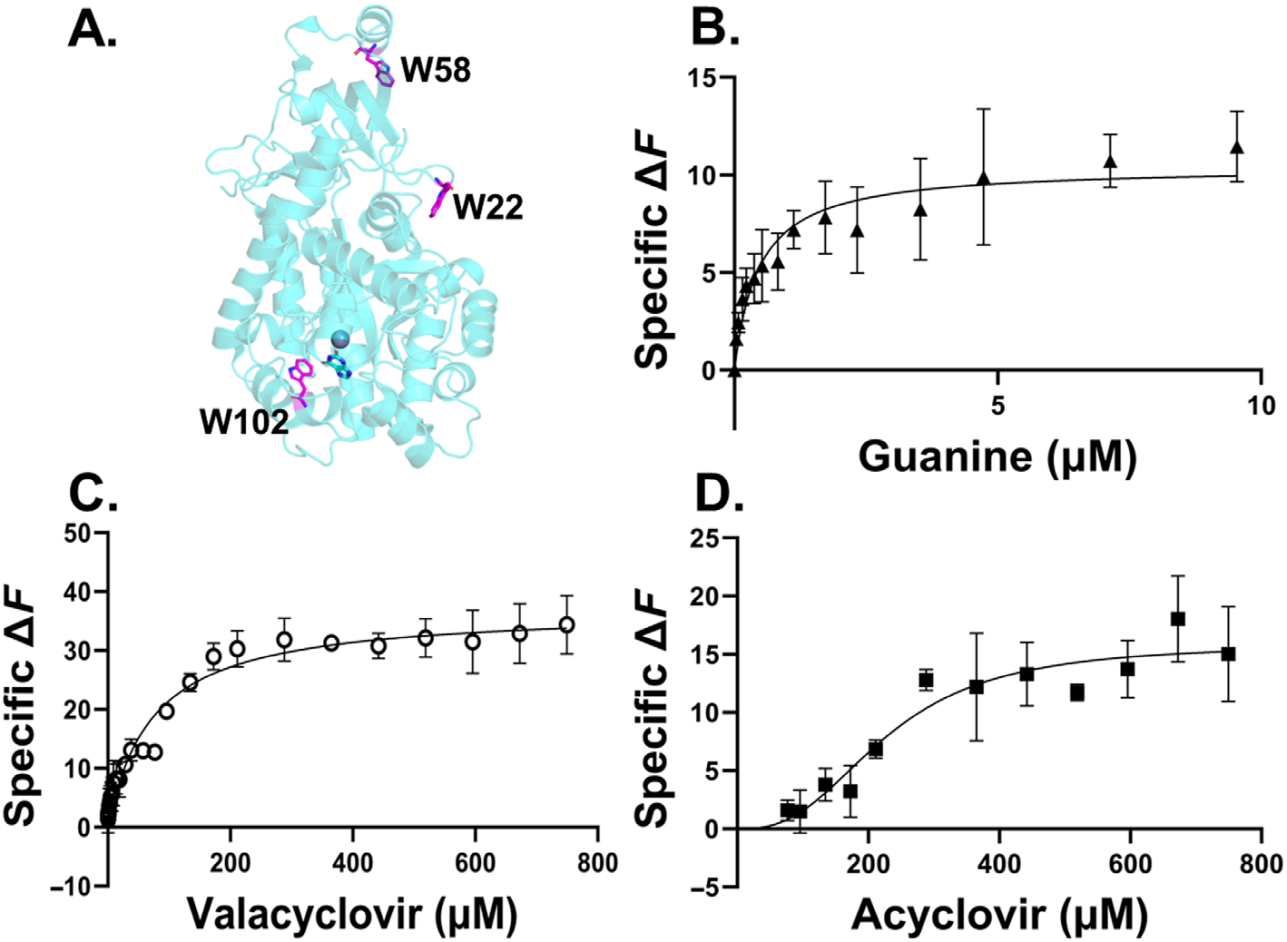
Determination of the dissociation constants (*K*_D_) of guanine, acyclovir, and valacyclovir from cypin. (A) Model of human cypin (PDB: 2UZ9, cyan) with all tryptophan residues highlighted as purple. (B) Determination of the *K*_D_ of guanine by assaying change in tryptophan fluorescence. (C) Determination of the *K*_D_ of valacyclovir by assaying change in tryptophan fluorescence. (D) Determination of the *K*_D_ of valacyclovir by assaying change in tryptophan fluorescence. Data points for guanine and valacyclovir were best fit with typical Michaelis–Menten kinetics while data for acyclovir were best fit using allosteric sigmoidal fit and demonstrate positive cooperativity (*h* = 1.19). Error bars represent mean ± SD.

The presence of tryptophan (W102) in the active site of cypin allows for the use of intrinsic tryptophan fluorescence for the determination of the dissociation constant of cypin ligands (Figure 2A). Given that the structures of guanine, acyclovir, and valacyclovir are based on a guanine motif but the structures vary in size, we sought to rank the *K*_D_ values of these molecules using changes to tryptophan fluorescence in cypin. Data for guanine were best fit with typical Michaelis–Menten kinetics, and we determined the *K*_D_ value for guanine as 0.598 ± 0.140 μM, which was the lowest value from the three ligands assayed (Figure 2B and Table 2). We determined the *K*_D_ for valacyclovir to be 74.4 ± 35.5 μM, more than 10 times higher than that of guanine, demonstrating lower affinity (Figure 2C). We found that acyclovir, which lacks the valine motif of valacyclovir, has a three‐fold higher *K*_D_ than that of valacyclovir, with a *K*_D_ value of 227.4 ± 18.7 μM (Figure 2D). Surprisingly, the data for acyclovir were best fit using specific binding with hill slope (*h* = 2.69). Unexpectedly, cooperative binding of any compound has not yet been reported for guanine deaminases in the amidohydrolase superfamily.

**TABLE 2:** *K*_D_ and *B*_max_ values of guanine and valacyclovir as determined by changes to intrinsic tryptophan fluorescence of cypin.

	Trial 1		Trial 2		Trial 3		Average (*x̄*)		Standard deviation (*σ*_*x*_)	
	*K*_D_ (μM)	*B*_max_ (|Δ*F*|)	*K*_D_ (μM)	*B*_max_ (|Δ*F*|)	*K*_D_ (μM)	*B*_max_ (|Δ*F*|)	*K*_D_ (μM)	*B*_max_ (|Δ*F*|)	*K*_D_ (μM)	*B*_max_ (|Δ*F*|)
Guanine	0.451	13.0	0.730	11.1	0.614	10.6	0.598	11.6	0.140	1.27
Valacyclovir	41.5	30.8	69.6	36.1	112	44.8	74.4	37.22	35.5	7.07
Acyclovir	247.9	20.1	222.9	15.5	211.4	14.4	227.4	16.7	18.7	3.02

### 3.3 Molecular Dynamics Simulations

**FIGURE 3:**
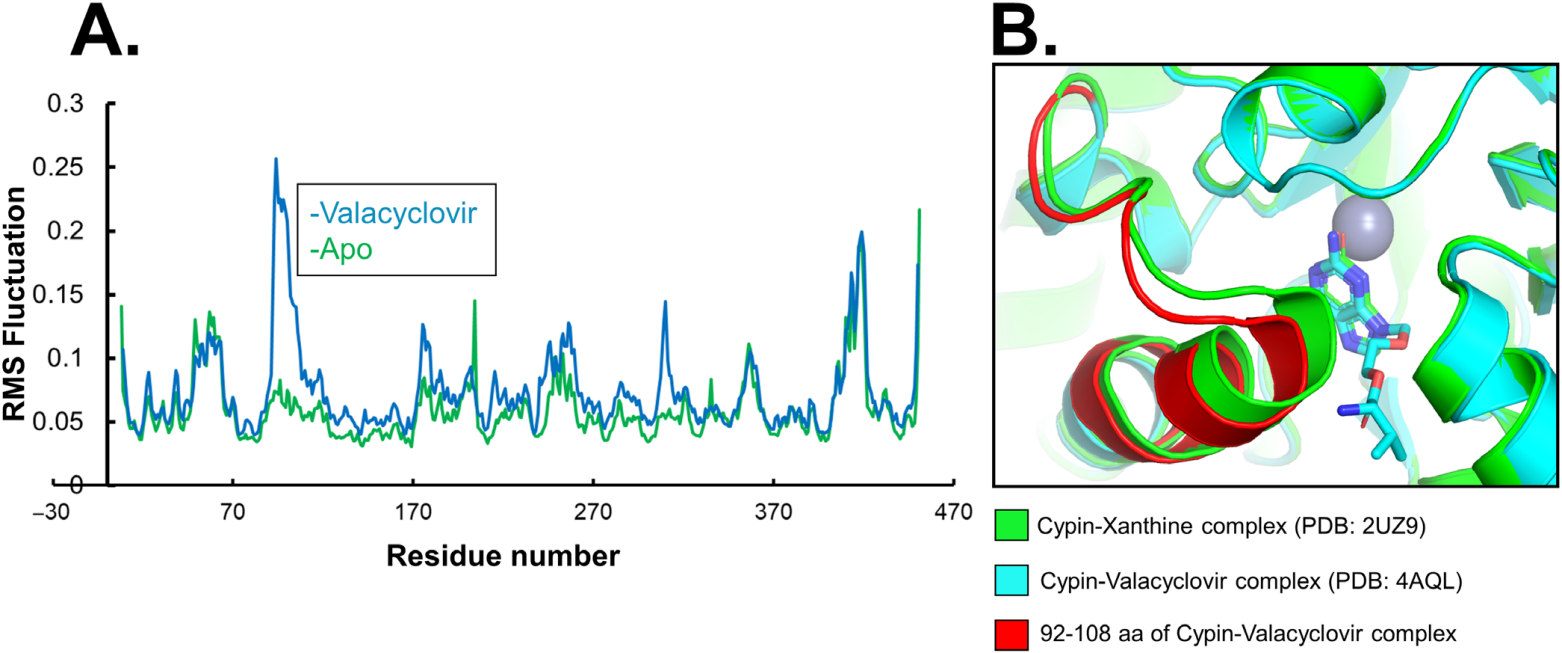
Molecular dynamics simulation demonstrates that valacyclovir changes the structure of cypin. (A) Comparison of the root mean square fluctuation of the residues of the valacyclovir‐bound (PDB: 4AQL, blue) and the structure of the cypin with xanthine removed (PDB: 2UZ9, green). (B) Alignment of the valacyclovir‐bound (cyan) and the apo structure of cypin (green).

To better understand the dynamics of the complex of valacyclovir and cypin, we compared the root mean square fluctuation (RMSF) of the residues of a valacyclovir‐bound cypin model (PDB: 4AQL) and an apo structure model of cypin with all ligands removed (PDB: 2UZ9) during a 10 ns molecular dynamics simulation. We reasoned that comparison of the movement of each amino acid, as reflected in the RMSF, may identify amino acids in the bound structure versus the apo structure that are involved in binding to larger guanosine molecules. The greatest difference in RMSF values was observed in amino acids 92–108 of the valacyclovir‐bound model, which implies that this region is more flexible when valacyclovir is bound (Figure 3A). Moreover, valacyclovir binding to cypin causes a 2.1 Å shift in the second alpha helix, using P98 as a reference point, compared to the apo cypin structure (Figure 3B).

### 3.4 Valacyclovir Is a Substrate of Cypin

**FIGURE 4:**
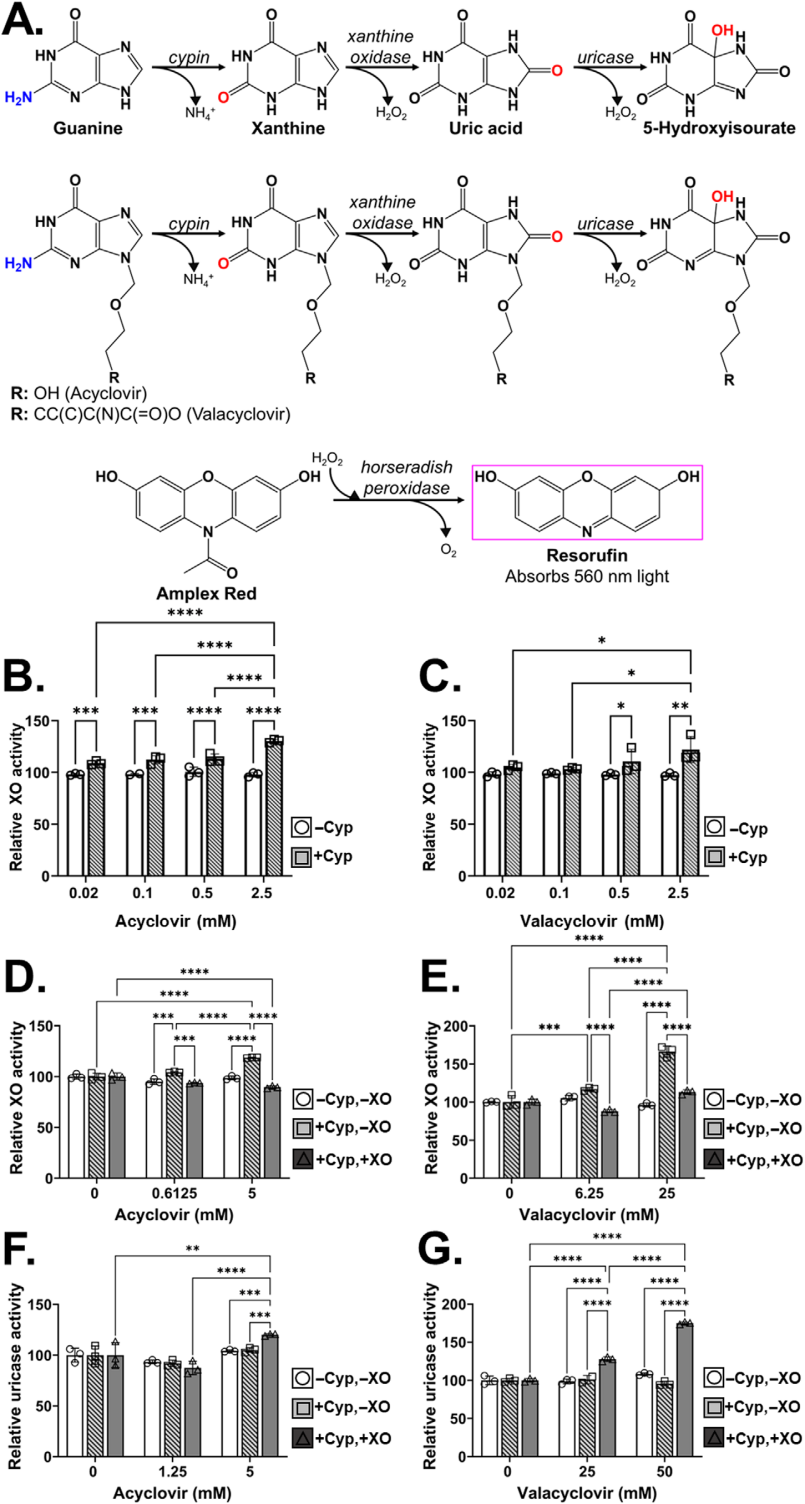
Guanosine analogs are substrates of the purine metabolism pathway only in the presence of cypin. (A) Pathway of guanosine analog metabolism tested. (B, C) Indicated concentrations of acyclovir and valacyclovir and were preincubated with 1 μM cypin for 4 h at 37°C in 100 mM Tris–HCl, 150 mM NaCl prior to assay. Xanthine oxidase activity was determined by Amplex Red assay. (D, E) Indicated concentrations of acyclovir and valacyclovir were incubated in the absence of cypin and xanthine oxidase (−Cyp, −XO), cypin only (+Cyp, −XO), or both cypin and xanthine oxidase (+Cyp, +XO) for 6 h at 37°C. Xanthine oxidase activity was determined by Amplex Red assay. (F, G) Indicated concentrations of acyclovir and valacyclovir were preincubated in the absence of cypin and xanthine oxidase (−Cyp, −XO), cypin only (+Cyp, −XO), or both cypin and xanthine oxidase (+Cyp, +XO) for 6 h at 37°C prior to assay. Uricase activity was determined by Amplex Red assay. *p < 0.05, **p < 0.01, ***p < 0.001, ****p < 0.0001 as determined by one way ANOVA followed by Tukey's multiple comparisons test versus vehicle. *n* = 3 independent trials. Cyp, cypin; XO, xanthine oxidase. Error bars represent mean ± SD.

To test whether enzymes in the purine metabolism pathway, specifically xanthine oxidase and uricase, can process acyclovir or valacyclovir after they are deaminated by cypin, we used an Amplex Red assay to measure peroxide, the byproduct of xanthine oxidase and uricase activity (Figure 4A). First, we performed reactions containing either acyclovir or valacyclovir in the presence of cypin and xanthine oxidase. We observed that xanthine oxidase activity is significantly increased above baseline when cypin is present (Figure 4B,C) after a 4‐h preincubation period with acyclovir or valacyclovir, suggesting that xanthine oxidase does not act directly on these analogs but instead acts on cypin‐mediated deaminated products. To confirm that the signal in the Amplex Red assay is not due to activity other than that of xanthine oxidase, such as exogenous peroxidases, we preincubated the analogs with either no enzyme, cypin alone, or both cypin and xanthine oxidase for 6 h prior to our assay for xanthine oxidase activity. Given the sensitivity of the Amplex Red assay and relatively higher *K*_m_ of valacyclovir compared to the *K*_m_ of guanine, we allowed the reaction to proceed for ≥2 h. Indeed, increased xanthine oxidase activity is only observed when cypin alone is present in the assay and not when the deaminated product has already been oxidized by xanthine oxidase (Figure 4D,E), supporting the idea that it is the presence of xanthine oxidase, and not another contaminating source of peroxide or enzyme from our purified recombinant cypin or drug stocks, that is, responsible for the signal. To determine whether uricase can act on the products of cypin and xanthine oxidase, we performed a similar experiment with preincubation but instead assessed uricase activity. As expected, an increase in uricase activity is only observed when both cypin and xanthine are present in the preincubation reaction (Figure 4F,G). Taken together, our data support the idea that cypin, xanthine oxidase, and uricase act sequentially on acyclovir and valacyclovir as they do on endogenous substrate guanine.

**FIGURE 5:**
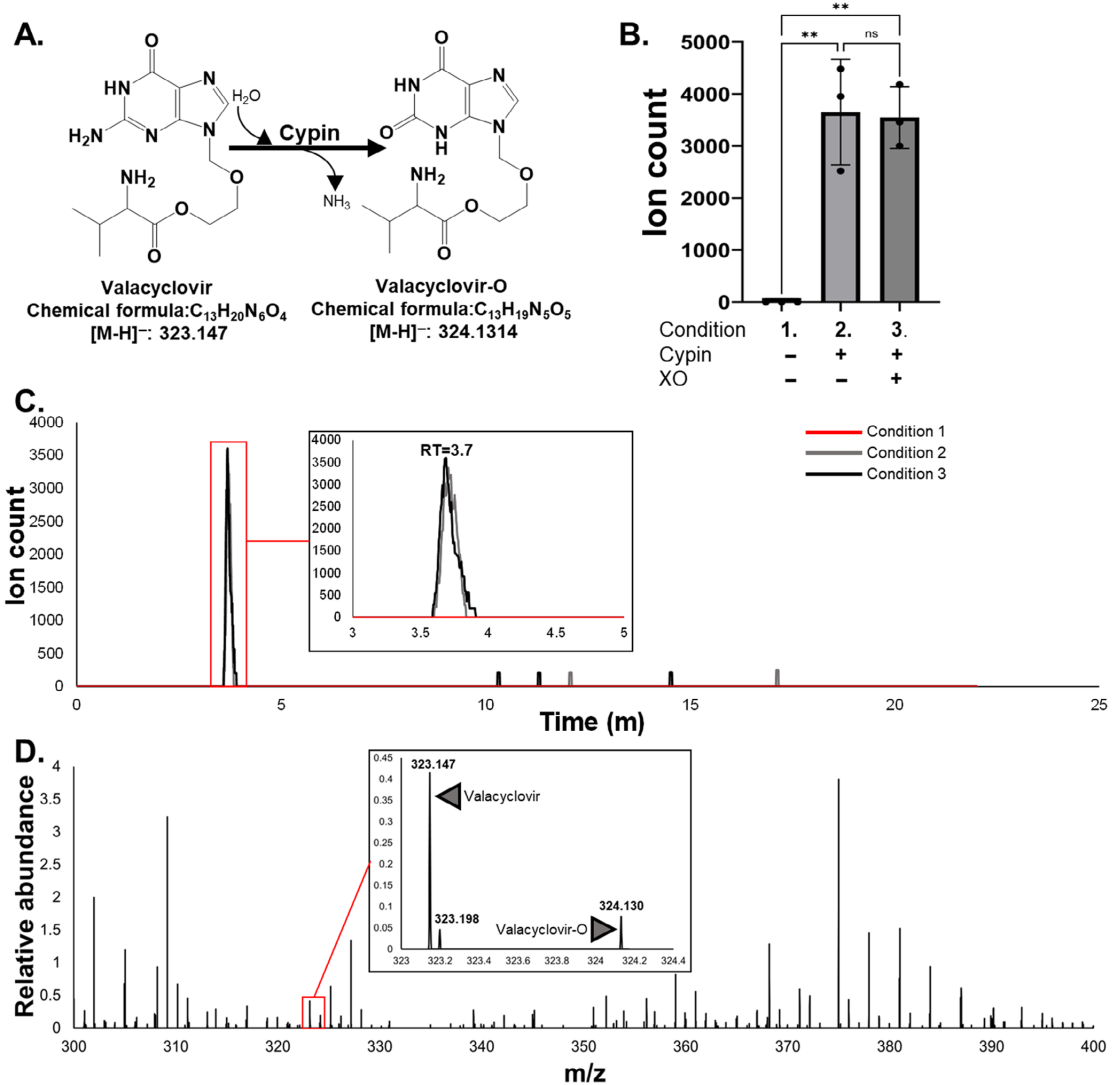
LC–MS detection of the deaminated valacyclovir by cypin. (A) Mass charge values of valacyclovir and deamination product of valacyclovir (valacyclovir‐O) using negative ion mode. (B) Total ion counts for three trials of three conditions (1; no enzyme, 2; only cypin, and 3; both cypin and xanthine oxidase). (C) Chromograms of three representative trials of condition 1 (red), condition 2 (gray), and condition 3 (black). The largest apex from the chromatogram was highlight in red and blown up. (D) Single mass spectrum from a single trial from condition 3 and the peaks corresponding to valacyclovir and deaminated valacyclovir are highlighted in red and blown up. **p < 0.01 as determined by one way ANOVA followed by Tukey's multiple comparisons test versus vehicle. *n* = 3 independent trials. Cyp, cypin; XO, xanthine oxidase. Error bars represent mean ± SD.

Using high‐resolution accurate mass (HRAM) LC–MS, we sought to directly detect the production of cypin‐promoted deamination of valacyclovir (Figure 5A). We observed ions associated with deaminated valacyclovir catalyzed in conditions where cypin was present (Figure 5B,C). The mass spectrum of this reaction demonstrates a peak corresponding to the predicted mass/charge ratio of deaminated valacyclovir (Figure 5D). However, while we did not detect ions corresponding to the mass/charge ratio of a xanthine oxidase‐mediated oxidization product of valacyclovir, we were able to directly measure the presence of a deaminated valacyclovir intermediate within 2 h at ~0.1% relative abundance.

## 4 Discussion and Conclusions

In the current study, we show that valacyclovir, and its active product acyclovir, can be deaminated by the *R. norvegicus* ortholog of the guanine deaminase cypin. The rat and human orthologs of cypin share 90.3% identity in primary structure, and thus, they most likely have similar structures [3]. We also show that concentrations of valacyclovir or acyclovir up to 2.5 mM do not attenuate the rate of detected deamination, suggesting that future cypin inhibitors should be developed to have greater affinity than guanine to be viable inhibitors. Interestingly, valacyclovir binds to cypin better than acyclovir, although valacyclovir is a prodrug and is designed to be efficiently hydrolyzed (~95%) by esterases present in the intestinal wall, gut lumen, and liver [18, 19]. Thus, the interaction between cypin and these guanosine analogs would primarily occur between cypin and the metabolized prodrug. We also demonstrate that the deaminated products of valacyclovir and acyclovir can proceed through the guanine degradation pathway as substrates of xanthine oxidase. Furthermore, the binding of valacyclovir to cypin causes a conformational change with movement of the second alpha helix of cypin away from the globular structure. This conformational change to cypin is unique to valacyclovir and demonstrates that cypin can bind ligands larger than nucleobases. Specifically, valacyclovir stabilizes cypin in the protein thermal shift assay, highlighting that small molecules can be developed can to target cypin. Taken together, our data demonstrate a previously unknown pathway of valacyclovir and acyclovir metabolism.

The binding of valacyclovir to guanine deaminase, that is, cypin, was previously reported [14]; however, the fact that it is deaminated by cypin was not known. Although this is an unexpected finding, there is precedence that deaminases can act on drugs. Specifically, cytidine deaminase degrades the cancer drug gemcitabine in the liver [20]. Viramidine, an antiviral drug, is converted to ribavirin and then to ICN3297, an inactive metabolite, by adenosine deaminase [21]. Additionally, deamination of prodrugs can result in activation. Deamination of the prodrug 2,6‐diamino‐9‐(2‐hydroxyethoxymethyl)purine by adenosine deaminase results in the active acyclovir metabolite [22]. Similarly, xanthine oxidase oxidizes the prodrug 6‐deoxyacyclovir to acyclovir [23]. Since cypin is highly expressed in the liver [6], where drug metabolism occurs, understanding the deamination pathways of prodrugs, and resulting active drugs, is needed for proper dosing and efficacy.

We determined the *K*_m_ value of guanine for purified recombinant guanine deaminase, cypin, from *R. norvegicus* as 0.120 ± 0.023 mM, which is consistent with previous reports of rat guanine deaminase purified from rat brain [24] but 10‐fold higher than what we observed for rabbit guanine deaminase [25]. Using a similar method to ours, *K*_m_ for the *S. cerevisiae* ortholog of guanine deaminase was calculated to be 2‐fold less than the value reported here for *R. norvegicus* cypin [26]. Interestingly, the reported *K*_m_ value for the human ortholog is 9.5 μM guanine, which was determined using a similar peroxidase‐dependent assay, demonstrating variability in enzyme efficiency between species. This difference in *K*_m_ values may be due to the fact that the cypin protein is not identical between mammalian orthologs. We also calculated specificity constants and found a 422 ± 56 fold higher constant for guanine than for valacyclovir, suggesting that while the turnover between the two substrates is equal when saturated, guanine is more efficiently processed than valacyclovir due to a higher affinity of cypin for guanine.

To our knowledge, we are the first to report *K*_D_ values of guanine, acyclovir, and valacyclovir for cypin. We used changes to tryptophan fluorescence in cypin to generate *K*_D_ values for guanine, acyclovir, valacyclovir. We hypothesized that valacyclovir would have greater affinity for cypin than acyclovir given the hydrophobic contacts from the additional valine motif in valacyclovir. Indeed, valacyclovir demonstrates three‐fold greater affinity for cypin. However, both valacyclovir and acyclovir have lower affinity for cypin compared to guanine, suggesting that larger guanosine analogs may induce strain in cypin when bound. This reinforces the idea that while cypin is more efficient at processing guanine than the larger guanosine analogs, the larger molecules do demonstrate affinity for cypin at amino acids just outside the site of catalysis. Regardless, the increase in the affinity of valacyclovir compared to acyclovir to cypin demonstrates that amino acids adjacent to the active site of cypin can bind ligands to cypin.

To our knowledge, we are the first report that cypin can process guanosine analogs; however, it is clear that valacyclovir is less efficiently processed than guanine, according to the specificity constants. Data from LC–MS directly confirms that the deamination of valacyclovir is indeed cypin‐dependent. We may not observe the xanthine oxidase‐dependent product due to the short time span of the assay and the low abundancy of the deaminated substrate. Reactions for LC–MS were completed with 2.5 mM valacyclovir concentration, which is below the Km, potentially limiting available substrates for xanthine oxidase. It is also possible that xanthine oxidase also acts on the deaminated valacyclovir product less efficiently than its endogenous substrate, xanthine. Furthermore, molecular dynamics data suggest that guanosine analogs induce more movement in the 92–108 amino acid region of cypin when bound compared to the movement when smaller nucleobases are bound to cypin. This amino acid region is involved in contributing hydrophobic contacts for the guanine base in the active site and we rationalize that displacement of this region impacts enzymatic efficiency. However, given the similar *V*_max_ values determined in guanine and valacyclovir saturation experiments, this displacement does not appear to be detrimental for the turnover of these analogs by cypin and is not sufficient for inhibition of cypin. The potential toxicity of the deaminated valacyclovir or the further oxidized variants is unknown and should be considered when designing cypin inhibitors.

One challenge of using guanosine analogs as cypin inhibitors is to ensure that the affinities of the inhibitors are higher than the affinity of guanine. However, comparison of the affinities of valacyclovir and acyclovir versus guanine for cypin highlights the fact that amino acids just outside of the active site can participate in ligand binding. Modification of the guanosine analog structure to a non‐metabolizable molecule may offer a promising lead for new cypin inhibitors. Moreover, future cypin inhibitors may contain planar, aromatic, or nucleobase analogs that mimic guanine but exclude the amine group, such as 6‐chloropurine or 6‐mercaptopurine. Additionally, future inhibitors may target amino acids that are near the active site, such as Leu‐100, Leu‐216, Asp‐246, and Thr‐176. The moieties of future inhibitors that target these areas should avoid bulky structures to accommodate the expanded hydrophobic cavity near the active site. Our studies suggest careful consideration of the possibility that therapeutics based on purine structures may be inactivated by cypin, decreasing inhibitory efficacy.

In humans, the final product of purine metabolism, uric acid, is a contributing factor in the progression of hyperuricemia and cardiovascular disease [27, 28]. Additionally, our group recently reported that treatment of mice with spinal cord‐induced neuropathic pain with intrathecal administration of a cypin inhibitor attenuates neuropathic pain [29]. Thus, development of effective cypin inhibitors may be helpful to multiple patients.

## Author Contributions

**Keith R. Lange:** conceptualization, methodology, software, formal analysis, data curation, visualization, investigation, writing – original draft, writing – review and editing. **Noor Rasheed:** investigation. **Xiaoyang Su:** methodology, writing – review and editing. **M. Elena Diaz‐Rubio:** methodology, data curation, formal analysis, visualization, investigation, writing – review and editing. **Bonnie L. Firestein:** conceptualization, methodology, writing – original draft, writing – review and editing, funding acquisition, project administration.

## Conflicts of Interest

The authors declare no conflicts of interest.

## Data Availability

The data that support the findings of this study are available from the corresponding author upon reasonable request.
